# Changes in metabolic syndrome status and risk of laryngeal cancer: A nationwide cohort study

**DOI:** 10.1371/journal.pone.0252872

**Published:** 2021-06-07

**Authors:** Hyun-Bum Kim, Geun-Jeon Kim, Kyung-do Han, Young-Hoon Joo

**Affiliations:** 1 Department of Otolaryngology-Head and Neck Surgery, College of Medicine, The Catholic University of Korea, Seoul, Korea; 2 Department of Biostatistics, College of Medicine, The Catholic University of Korea, Seoul, Korea; German Centre for Neurodegenerative Diseases Site Munich: Deutsches Zentrum fur Neurodegenerative Erkrankungen Standort Munchen, GERMANY

## Abstract

**Background:**

Whether dynamic changes of metabolic syndrome (MetS) affects the subsequent laryngeal cancer occurrence remains unknown.

**Objective:**

This study investigated the effects of changes of MetS on the incidence of laryngeal cancer due to a lack of knowledge regarding the development of MetS in Korean population.

**Methods:**

A total of 6,757,048 individuals who received national health checkup in 2009 and follow-up health examination in 2011 were analyzed and followed up until 2018. MetS status included the following categories: MetS-chronic (n = 941,609), MetS-developed (n = 614,229), MetS-recovery (n = 455,835), and MetS-free (n = 4,745,375).

**Results:**

With a median follow-up duration of 6.403 years, 1,350 subjects were newly diagnosed with laryngeal cancer. Compared to participants without MetS, adjusted hazard ratios (HR) (95% confidence interval) for those with MetS were 1.320 (1.17–1.489) for laryngeal cancer. The HR of laryngeal cancer was found to be increased with increasing number of MetS components. The MetS-developed group had a significantly higher risk of laryngeal cancer than the MetS-free group (HR: 1.296; 95% CI: 1.093–1.537). The MetS-recovery group within two years also had an increased risk of laryngeal cancer compared with the MetS-free group (HR: 1.220; 95% CI: 1.008–1.476). Among MetS components, abdominal obesity had the highest risk of laryngeal cancer (HR: 1.374; 95% CI: 1.123–1.681).

**Conclusion:**

Changes in MetS status were associated with the risk of laryngeal cancer. Results of this study have implications for etiological investigations and prevention strategies.

## Introduction

Laryngeal cancer belongs to head and neck malignancies. About 177,422 new cases of laryngeal cancer were diagnosed in 2018 worldwide [1]. In Korea, there are about 1,160 new cases every year. The average incidence was 2.3 per 100,000 from 2008 to 2017 [2]. Several studies about the etiology have shown that smoking, alcohol consumption, and metabolic abnormalities are related to the development of laryngeal cancer [3, 4].

Metabolic syndrome (MetS) was first created by Haller and Hanefeld in 1975 and is a cluster of risk factors for cardiovascular disease that occur together more often than by chance alone [5–7]. The main risk factors for the development of MetS are physical inactivity and a high-fat and high-carbohydrate diets, which contribute to two key clinical characteristics: central obesity and insulin resistance [8]. The number of people with MetS is steadily increasing. About one third of US adults have MetS [9].

Some studies have shown that MetS increases the risk of several cancer, including liver, colorectal, bladder in men, and endometrial, pancreatic, breast postmenopausal, rectal, colorectal in women [10]. Individual components of MetS are known as risk factors for incident cancer. However, it is still unclear how clustering of these components is related to the development and progression of tumors. We have previously reported the association of MetS with laryngeal cancer incidence [4]. MetS is a treatable disease. Management of each MetS component has proven to be effective in reducing the risk for major adverse cardiovascular events [11]. However, cancer risk after MetS improvement or development has not been reported yet. We hypothesized that persons with an altered MetS status or alterations in each MetS component would have an altered risk for laryngeal cancer. Thus, the objective of this study was to investigate the association between dynamic MetS status and laryngeal cancer risk.

## Materials and methods

### Ethics statement

This entire study was approved by the Institutional Review Board of the Bucheon St. Mary’s hospital (IRB File No. HC 19ZISI0122). All participants in this study signed an informed consent form.

### Data source

This study was based on the Korean National Health Insurance Service (KNHIS), a population-based cohort [12]. It offers free health check-up for office/non-office subscribers every two years and non-office workers every year. Dependents over the age of 40 also have free health check-up every two years. About 15 million people receive medical checkups every year. We included individuals older than 40 years who underwent KNHIS health checkup in 2009 and follow-up health examination in 2011. The development of laryngeal cancer was assessed until 2018 using KNHIS claims records during the study period. Diagnosis of laryngeal cancer was made using codes of the International Classification of Disease, Tenth Revision, Clinical Modification (ICD-10-CM): C320 Glottic cancer, C321 Supraglottic cancer, C32.2 Subglottic cancer, C323 Laryngeal cartilage cancer, C328 Overlapping sites of larynx cancer, and C329 Larynx cancer, unspecified.

### Study population

**Fig 1:**
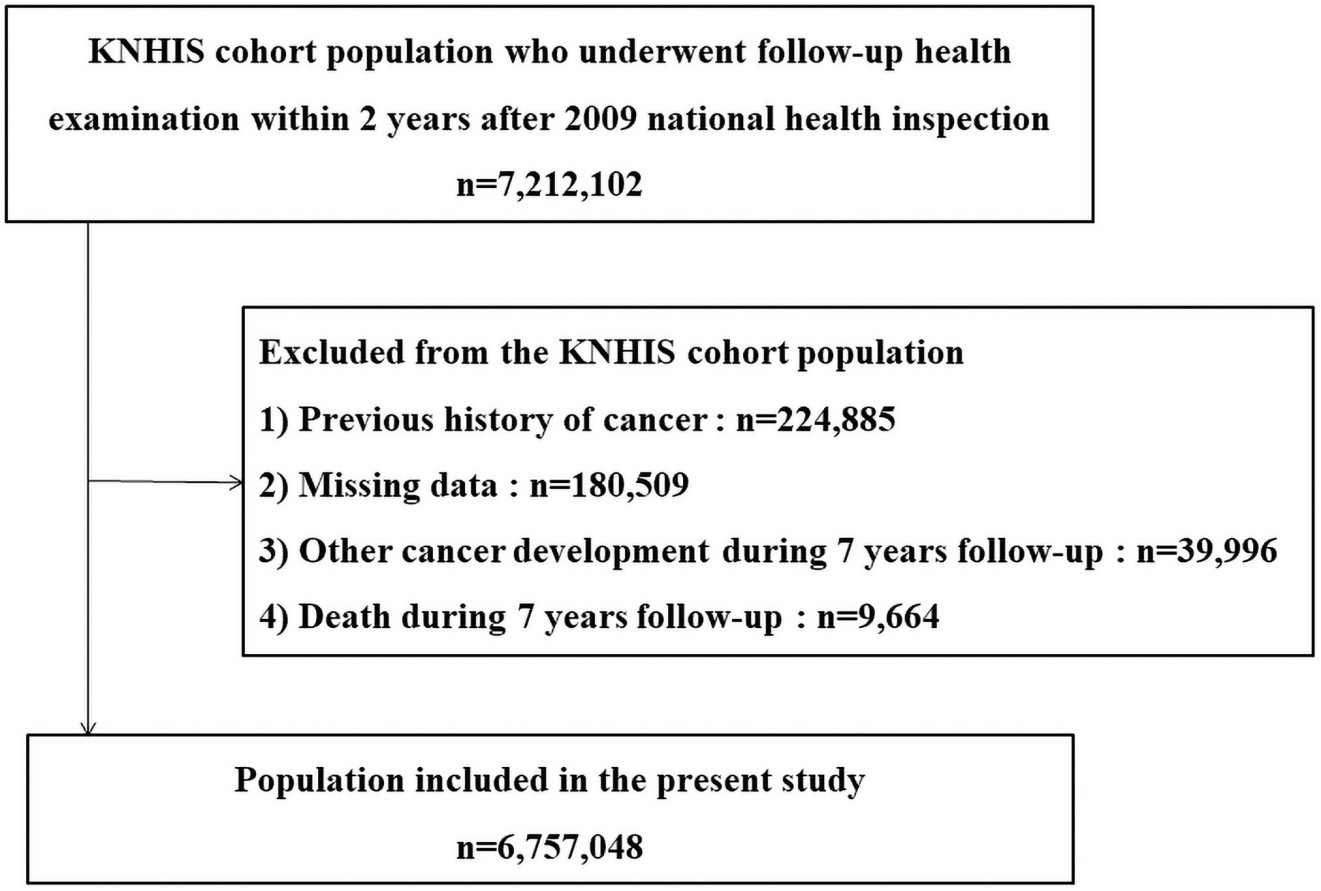
Study profile.

Patients were defined as having laryngeal cancer if they had admission records for primary laryngeal cancer in KNHIS data from 2011 to 2018 with a median follow-up duration of 6.403 years. Among 7,212,102 individuals with health examinations at both time points, 180,509 subjects with missing data and 224,885 subjects with previous history of cancer were excluded. We also excluded 2,520,651 who had been diagnosed with other cancers or died during the follow-up period. Finally, a total of 6,757,048 individuals were included in this study (Fig 1). The blood pressure (BP) measurement was taken according to the Korean Society of hypertension guidelines. BP was measured more than twice at 1- to 2-min intervals using a mercury or an automatic sphygmomanometer after anthropometric measurements with rest for 5 or more minutes in a quiet, appropriate environment. A positive antihypertensive medication history was defined as answering “Yes” to corresponding questions on the health screening questionnaire. Levels of fasting blood glucose (FBG), triglyceride (TG), total cholesterol, and high-density lipoprotein (HDL) cholesterol were also obtained. Medical history information and health-related behaviors were collected using standardized self-reporting questionnaires.

### Definition of MetS and study subgroups

The definition of MetS was based on the definition established by the joint interim statement of the International Diabetes Federation Task Force on Epidemiology and Prevention [13]. MetS should have at least three of the following five components: (1) central obesity (waist circumference (WC) ≥ 90 cm for men or ≥ 85 cm for women), (2) systolic blood pressure (BP) ≥ 130 mmHg and/or diastolic BP ≥ 85 mmHg) or the use of antihypertensive medication, (3) elevated fasting blood glucose (FBG) ≥ 100 mg/dL or the use of hypoglycaemic agent, (4) hypertriglyceridemia (TG) ≥ 150 mg/dL, and (5) low HDL-cholesterol levels < 40 mg/dL for men or < 50 mg/dL for women. Body mass index (BMI) was calculated as the weight in kilograms divided by the square of the height in meters (kg/m^2^). Subjects were subdivided according to two consecutive health checkups conducted every two years. Subjects who failed to meet the diagnostic criteria for MetS in both medical examinations were assigned to the “MetS-free group”. Subjects with MetS in both medical examinations were assigned to the “MetS-chronic group”. Those who developed MetS that did not exist in 2009 during medical examinations whose MetS lasted until the 2011 medical examinations were assigned to the “MetS-developed group”. The “MetS-recovery group” included individuals who had MetS that recovered during 2011 medical examinations.

### Statistical analysis

All statistical analyses were performed using SAS software, version 9.2 (SAS Institute, Cary, North Carolina, US). The reference characteristics were described with mean and standard deviation or percentage of categorical variables. Independent t-test (continuous variables) and Chi-square test (categorical variables) was used to compare the variables. The incidence of laryngeal cancer was calculated by dividing the number of patients by 1,000 person-years. Cox regression analysis was used to evaluate the correlation between different variables and the incidence of laryngeal cancer according to the MetS state and to obtain a risk ratio (HR) and 95% confidence interval (CI). We considered p-value < 0.05 to be statistically significant.

## Results

### Basic characteristics

Characteristics of this study population are shown in Table 1. With a median follow-up duration of 6.403 years, 1,350 subjects were newly diagnosed with laryngeal cancer. The mean age of patients with laryngeal cancer was significantly (p < 0.0001) higher than that of those without such cancer. The incidence of laryngeal cancer in men was significantly (p < 0.0001) higher than that in women. Current or ex-smokers and heavy drinkers was positively correlated with laryngeal cancer (p < 0.0001). Study participants with laryngeal cancer had significantly (p < 0.0001) higher mean waist circumferences, systolic and diastolic BPs, FBG levels, and fasting TG levels than those without laryngeal cancer. They also had higher rates of having MetS-chronic (25.19% vs. 13.93%), MetS-recovery (9.63% vs. 6.75%), and MetS-developed (13.04 vs. 9.09%) status.

**Table 1:** Analysis of factors potentially associated with laryngeal cancer (n = 6,757,048).

Parameter	Yes (n = 1,350)	No (n = 6,755,698)	P-value
Age (years)	61.35±9.38	48.51±13.43	<0.0001*
Gender (Male)	1,291(95.63%)	3,853,435(57.04%)	<0.0001*
Smoking status			<0.0001*
Non smoker	307(22.74%)	3,943,153(58.37%)	
Ex smoker	410(30.37%)	1,142,775(16.92%)	
Current smoker	633(46.89%)	1,669,770(24.72%)	
Drinking status			<0.0001*
Non drinker	488(36.15%)	3,390,478(50.19%)	
Mild drinker	654(48.44%)	2,874,117(42.54%)	
Heavy drinker	208(15.41%)	491,103(7.27%)	
Regular exercise	317(23.48%)	1,360,901(20.16%)	0.0024*
Diabetes	284(21.04%)	604,761(8.95%)	<0.0001*
Hypertension	681(50.44%)	1,851,895(27.41%)	<0.0001*
Dyslipidemia	366(27.11%)	1,358,466(20.11%)	<0.0001*
Body mass index (kg/m^2^)	23.73±2.9	23.73±3.15	0.3817
Waist circumference (cm)	84.36±7.96	80.27±8.92	<0.0001*
Systolic BP (mmHg)	127.61±14.94	122.23±14.65	<0.0001*
Diastolic BP (mmHg)	78.62±9.29	76.32±9.87	<0.0001*
Fasting glucose (mg/dL)	104.47±29.69	96.56±21.94	<0.0001*
HDL cholesterol (mg/dL)	53±19.45	55.38±19.42	<0.0001*
Triglyceride (mg/dL)	127.19(123.58–130.91)	111.72(111.68–111.77)	<0.0001*
Metabolic syndrome			<0.0001*
MetS-free	704(52.15%)	4,744,671(70.23%)	
MetS-developed	176(13.04%)	614,053(9.09%)	
MetS-recovery	130(9.63%)	455,705(6.75%)	
MetS-chronic	340(25.19%)	941,269(13.93%)	

Values are mean ± SE or % ± SE.

* Significant at p<0.05.

### MetS and risk of laryngeal cancer

Unadjusted and multivariable-adjusted HRs of laryngeal cancer for each individual component of MetS are shown in Table 2. Age, gender, smoking status, alcohol intake, exercise, and BMI-adjusted hazard ratio of laryngeal cancer was 1.32 times higher for participants with MetS than for those without MetS. The multivariable model shows that elevated FBG (HR: 1.252; 95% CI: 1.067–1.445), high WC (HR: 1.242; 95% CI: 1.067–1.445), elevated TG (HR: 1.166; 95% CI: 1.044–1.302), elevated BP (HR: 1.161; 95% CI: 1.035–1.302), and low HDL cholesterol (HR: 1.146; 95% CI: 1.017–1.290) were significantly associated with an increased hazard of larynx cancer. Furthermore, the number of MetS components positively associated with laryngeal cancer (HR: 1.742; 95% CI: 1.288–2.356 for five MetS components). HRs of laryngeal cancer increased with increasing number of MetS components.

**Table 2:** Multivariate Cox proportional hazard model for incidence of larynx cancer according to the metabolic syndrome component.

Parameter	No of patients	Person-years	Incidence rates per 1000	Hazard Ratio (95% confidence interval)		
				Model 1	Model 2	Model 3
Metabolic syndrome						
Yes	516	9,891,381	0.05	2.065(1.850–2.305)	1.236(1.106–1.382)	1.320(1.17–1.489)
No	834	33,118,165	0.03	1 (reference)	1 (reference)	1 (reference)
High waist circumference						
Yes	356	8,523,109	0.04	1.447(1.282–1.633)	1.08(0.957–1.219)	1.242(1.067–1.445)
No	994	34,486,437	0.03	1 (reference)	1 (reference)	1 (reference)
High glucose						
Yes	675	13,571,690	0.05	2.166(1.947–2.410)	1.253(1.125–1.396)	1.252(1.123–1.396)
No	675	29,437,855	0.02	1 (reference)	1 (reference)	1 (reference)
High triglyceride						
Yes	653	15,819,068	0.04	1.608(1.445–1.789)	1.198(1.077–1.333)	1.166(1.044–1.302)
No	697	27,190,478	0.03	1 (reference)	1 (reference)	1 (reference)
Low HDL cholesterol						
Yes	434	12,104,486	0.04	1.205(1.075–1.351)	1.093(0.973–1.227)	1.146(1.017–1.290)
No	916	30,905,060	0.03	1 (reference)	1 (reference)	1 (reference)
High blood pressure						
Yes	681	12,842,601	0.05	2.383(2.142–2.652)	1.116(0.998–1.247)	1.161(1.035–1.302)
No	669	30,166,944	0.02	1 (reference)	1 (reference)	1 (reference)
No of MetS components						
0	205	13,587,248	0.02	1 (reference)	1 (reference)	1 (reference)
1	314	11,285,445	0.03	1.842(1.545–2.197)	1.138(0.954–1.358)	1.158(0.969–1.383)
2	315	8,245,470	0.04	2.527(2.120–3.013)	1.251(1.047–1.493)	1.309(1.091–1.570)
3	275	5,588,062	0.05	3.252(2.714–3.896)	1.423(1.185–1.709)	1.544(1.273–1.872)
4	175	3,196,214	0.06	3.613(2.953–4.421)	1.384(1.128–1.699)	1.559(1.254–1.938)
5	66	1,107,104	0.06	3.930(2.978–5.186)	1.434(1.084–1.898)	1.742(1.288–2.356)

Model 1: Unadjusted.

Model 2: Adjusted for age, gender.

Model 3: Adjusted for age, gender, smoking status, alcohol intake, exercise, and body mass index.

### Risk of laryngeal cancer according to MetS changes

Numbers of patients with laryngeal cancer in MetS-free, MetS-developed, MetS-recovery, and MetS- chronic groups were 704, 176, 130, and 340, respectively. Table 3 presents data on the relationship between laryngeal cancer risk and MetS changes according to logistic regression models. In our regression analysis, the MetS-chronic group (HR: 1.420; 95% CI: 1.229–1.641) had the highest the risk of laryngeal cancer, followed by the MetS-developed group (HR: 1.296; 95% CI: 1.093–1.537) and the MetS-recovery group (HR: 1.220; 95% CI: 1.008–1.476) after adjusting for age, gender, smoking status, alcohol intake, exercise, and BMI. We also investigated the association of each component with the risk of laryngeal cancer. For WC, HR for laryngeal cancer was the highest in individuals with WC-chronic group (HR: 1.374; 95% CI: 1.123–1.681). FBG-developed and FBG-chronic groups were associated with laryngeal cancer, with HR (95% CI) of 1.196 (1.017–1.407) and 1.359 (1.194–1.548). Compared to the TG-free group, other groups showed a higher risk of laryngeal cancer (HR: 1.211; 95% CI: 1.027–1.428 in the TG-developed, HR: 1.2142; 95% CI: 1.024–1.439 in the TG-recovery, HR: 1.233; 95% CI: 1.081–1.406 in the TG-chronic). For HDL cholesterol, laryngeal cancer was only associated with HDL developed group (HR: 1.192; 95% CI: 1.014–1.401). The risk of laryngeal cancer was associated with the BP-chronic group (HR: 1.199; 95% CI: 1.054–1.364).

**Table 3:** Multivariate Cox proportional hazard model for larynx cancer risk according to changes in metabolic syndrome and components.

Parameter	No of patients	Person -years	Incidence rates per 1000	Hazard Ratio (95% confidence interval)		
				Model 1	Model 2	Model 3
Metabolic syndrome						
MetS-free	704	30,222,645	0.02	1(reference)	1(reference)	1(reference)
MetS-developed	176	3,908,438	0.05	1.928(1.634–2.274)	1.225(1.037–1.445)	1.296(1.093–1.537)
MetS-recovery	130	2,895,519	0.05	1.924(1.596–2.320)	1.162(0.963–1.402)	1.220(1.008–1.476)
MetS-chronic	340	5,982,942	0.07	2.431(2.136–2.767)	1.288(1.129–1.469)	1.420(1.229–1.641)
Waist circumference						
WC-free	867	31,742,849	0.03	1(reference)	1(reference)	1(reference)
WC-developed	127	3,157,773	0.04	1.470(1.220–1.771)	1.191(0.989–1.435)	1.374(1.123–1.681)
WC-recovery	127	2,743,588	0.05	1.691(1.404–2.037)	1.210(1.004–1.459)	1.365(1.122–1.661)
WC-chronic	229	5,365,335	0.04	1.559(1.348–1.804)	1.062(0.918–1.230)	1.318(1.092–1.589)
Fasting blood glucose						
FBG-free	495	24,274,659	0.02	1(reference)	1(reference)	1(reference)
FBG-developed	210	5,694,326	0.04	1.807(1.538–2.124)	1.212(1.031–1.424)	1.196(1.017–1.407)
FBG-recovery	180	5,163,196	0.04	1.709(1.441–2.027)	1.175(0.990–1.394)	1.162(0.979–1.378)
FBG-chronic	465	7,877,364	0.06	2.890(2.547–3.280)	1.355(1.192–1.540)	1.359(1.194–1.548)
Triglyceride						
TG-free	516	22,631,613	0.02	1(reference)	1(reference)	1(reference)
TG-developed	199	5,517,700	0.04	1.580(1.342–1.861)	1.239(1.052–1.460)	1.211(1.027–1.428)
TG-recovery	181	4,558,865	0.04	1.740(1.469–2.061)	1.250(1.055–1.481)	1.214(1.024–1.439)
TG-chronic	454	10,301,367	0.04	1.930(1.701–2.189)	1.275(1.124–1.446)	1.233(1.081–1.406)
HDL cholesterol						
HDL-free	778	26,600,773	0.03	1(reference)	1(reference)	1(reference)
HDL-developed	187	4,957,891	0.04	1.286(1.096–1.508)	1.145(0.976–1.344)	1.192(1.014–1.401)
HDL-recovery	138	4,304,286	0.03	1.094(0.913–1.311)	1.102(0.919–1.321)	1.129(0.940–1.355)
HDL-chronic	247	7,146,595	0.04	1.177(1.020–1.358)	1.082(0.936–1.252)	1.151(0.991–1.336)
Blood pressure						
BP-free	563	27,353,809	0.02	1(reference)	1(reference)	1(reference)
BP-developed	146	3,724,217	0.04	1.901(1.585–2.281)	1.121(0.933–1.345)	1.122(0.934–1.349)
BP-recovery	106	2,813,135	0.04	1.829(1.486–2.251)	1.082(0.879–1.333)	1.096(0.889–1.350)
BP-chronic	535	9,118,384	0.06	2.841(2.524–3.198)	1.134(1.000–1.285)	1.199(1.054–1.364)

Model 1: Unadjusted.

Model 2: Adjusted for age, gender.

Model 3: Adjusted for age, gender, smoking status, alcohol intake, exercise, and body mass index.

### MetS changes according to baseline characteristics

The association between the risk of laryngeal cancer and change of MetS according to baseline characteristics is shown in Table 4. In the MetS-chronic group, the risk of laryngeal cancer was associated with middle age, male, never or ex-smoker, none or mild alcohol drinker, and no dyslipidemia. However, MetS-chronic was correlated with laryngeal cancer regardless of regardless of the presence or absence of diabetes or hypertension. For the MetS-developed group, laryngeal cancer were associated with middle age, male, never smoker, none alcohol intake, no dyslipidemia, and low BMI. The risk of laryngeal cancer was also associated with the MetS-recovery group in male, never smoker, and no dyslipidemia.

**Table 4:** Analysis of factors potentially associated with laryngeal cancer.

Parameter	MetS-free	MetS-developed	MetS-recovery	MetS-chronic
Age (years)				
<40	1(reference)	1.837(0.383–8.819)	2.428(0.519–11.346)	2.607(0.493–13.795)
<65	1(reference)	1.284(1.032–1.598)	1.182(0.923–1.515)	1.468(1.219–1.767)
≥65	1(reference)	1.135(0.863–1.492)	1.096(0.810–1.483)	1.120(0.893–1.406)
Gender				
Male	1(reference)	1.308(1.099–1.557)	1.221(1.004–1.484)	1.449(1.251–1.680)
Female	1(reference)	1.123(0.478–2.641)	1.314(0.531–3.250)	0.990(0.474–2.067)
Smoking status				
Never smoker	1(reference)	1.634(1.146–2.330)	1.748(1.199–2.547)	1.877(1.390–2.534)
Ex-smoker	1(reference)	1.350(0.995–1.832)	1.247(0.883–1.762)	1.520(1.180–1.959)
Current smoker	1(reference)	1.144(0.888–1.474)	1.012(0.757–1.353)	1.191(0.956–1.484)
Alcohol intake				
None	1(reference)	1.376(1.038–1.824)	1.158(0.833–1.610)	1.498(1.182–1.898)
Mild	1(reference)	1.261(0.984–1.617)	1.241(0.945–1.629)	1.438(1.168–1.770)
Heavy	1(reference)	1.229(0.807–1.873)	1.273(0.805–2.011)	1.196(0.817–1.752)
Diabetes				
Yes	1(reference)	1.400(0.946–2.071)	0.988(0.602–1.621)	1.443(1.040–2.002)
No	1(reference)	1.184(0.974–1.440)	1.220(0.991–1.502)	1.252(1.045–1.499)
Hypertension (%)				
Yes	1(reference)	1.222(0.975–1.532)	1.093(0.824–1.449)	1.321(1.089–1.603)
No	1(reference)	1.172(0.875–1.571)	1.258(0.967–1.635)	1.389(1.044–1.849)
Dyslipidemia (%)				
Yes	1(reference)	1.016(0.729–1.416)	0.978(0.585–1.635)	1.250(0.941–1.661)
No	1(reference)	1.410(1.141–1.741)	1.255(1.021–1.542)	1.429(1.167–1.749)
Body mass index (kg/m^2^)				
<25	1(reference)	1.219(0.984–1.511)	1.166(0.921–1.474)	1.314(1.092–1.580)
≥25	1(reference)	1.372(1.031–1.827)	1.276(0.917–1.774)	1.446(1.145–1.824)

## Discussion

In Korea, according to Korea Central Cancer Registry, the average incidence of laryngeal cancer was 2.3 per 100,000 from 2008 to 2017 [2]. The gender ratio of males to females is 15:1 for those with of laryngeal cancer, indicating that it is more common in men. By age group, the incidence of laryngeal cancer was 33.5% for those in the 7^th^ decade, 29,9% for those in the 8^th^ decade, and 21.6% for those in the 5^th^ decade. Tobacco smoking and alcohol consumption are well known as risk factors of laryngeal cancer [14]. Tobacco smoking is the most powerful risk factor in any condition. In contrast, alcohol consumption is a significantly risk factor in smokers, but not in nonsmokers.

The prevalence of MetS is increasing. MetS has recently attracted attention as a risk factor for many cancers [3]. We previously reported the association with MetS and incidence of laryngeal cancer [4]. Study population with MetS had a 1.13-fold higher hazard of having larynx cancer than those without MetS. In the pathophysiologic course by which MetS causes cancer, peroxisome proliferator-activated receptors (PPARs) play an important role. They belong to the superfamily of nuclear hormone receptors. They affect the regulation of cancer cell growth. PPARs have three types. One of them is PPAR-gamma that can regulate immunity, inflammation, and growth of tumors in many organs including squamous cells.

In the present study, we investigated the correlation between dynamic changes of MetS and the incidence of laryngeal cancer. We found that the risk of laryngeal cancer varied significantly according to the dynamic MetS status of a nationwide population-based cohort. Compared to the MetS-chronic group (HR: 1.420), people who recovered from MetS (HR: 1.220) had a lower risk of developing laryngeal cancer. In addition, people who developed MetS (HR: 1.296) were at greater risk of developing laryngeal cancer than those who remained MetS-free. The associations remained significant even after adjustment for age, gender, smoking status, alcohol intake, exercise, and BMI. The benefit of recovering MetS, or harm associated with developing MetS, can occur regardless of the health-related behaviors, underlying obesity, or comorbidity burden. Health care providers may consider these consequences when planning a public health strategies to ease the burden of laryngeal cancer.

Compared to the case without MetS, the risk of laryngeal cancer was increased in those with MetS despite improvement of MetS. This shows that MetS is still a risk factor for laryngeal cancer, even if there is a recovery from MetS. Oh et al reported similar result about the MetS status and kidney cancer [15]. Patients with improved MetS within two years had increased risk of kidney cancer compared with those without MetS. This phenomenon might be correlated with metabolic memory. When hyperglycemia occurs, a series of intracellular protein reactions remain as memories and affect long-term complications. Over the last decade, follow-up of the original Diabetes Control and Complications Trial (DCCT) cohort has revealed that individuals in end of the initial DCCT phase have usual glycemic control and continue to have better outcomes for microvascular and macrovascular events, although they have been randomized initially to a more intensified insulin regimen [16, 17]. The underlying explanation for sustained effects of prior changes in glucose homeostasis with either improved or worse metabolic control at biochemical or molecular level remains to be elucidated. Many animal models have been developed to mimic metabolic memory. The molecular basis of oxidative stress and epigenetic mechanisms for modifying gene expression is fully explained [18]. There are evidence to support the fact that high glucose level can cause anomalies in post-translational modifications of histones, DNA methyltransferase, and miRNA levels. Aggregation of AGEs and oxidative stress are factors that can lead to metabolic memory in endothelial cells [19, 20].

In MetS components, hazard ratios of elevated BP and hyperglycemia increased in the order of MetS-free, MetS-recovery, MetS-developed, and MetS-chronic, with MetS-chronic having the highest hazard ratio of elevated BP and hyperglycemia. Several studies have reported that diabetes affects laryngeal cancer [21, 22]. In this context, the present study also revealed that diabetes elevated the incidence of laryngeal cancer. Hypertension is well-known as a factor that can elevate morbidity in laryngeal cancer. However, the relationship between hypertension and incidence of laryngeal cancer remains unclear. In the present study, hypertension elevated the incidence of laryngeal cancer in an unadjusted model (Model 1). However, in the model after adjusting for age, gender, smoking status, alcohol intake, exercise, and body mass index (Model 3), there was a weak correlation between hypertension and the incidence of laryngeal cancer. The pathophysiological mechanism by which hypertension affects the incidence of laryngeal cancer remains unclear. It needs further studies. Regarding other MetS components, abdominal obesity, hypertriglyceridemia, and low HDL-cholesterol levels were associated with obesity.

Many studies have reported the relationship between obesity and cancer risk [23–25]. In the present study, obesity as one of MetS components had the highest risk for laryngeal cancer. Obesity means storage of excess nutrients. People save excess nutrients as adipocytes in white adipose tissues [26]. Adipocytes can release lots of cytokines, hormones, and growth factors. As a result, obesity causes an increase of adipocytes known to secrete many cytokines and hormones, resulting in chronic low-grade inflammation and several changes of hormone axis. Both chronic low-grade inflammation and several changes of hormone axis can induce changes in the activation of macrophage and compositions of T cells. Such changes might eventually lead to carcinogenesis.

This study has some limitations. First, we examined changes of MetS at health screenings at only a two-year interval. Thus, we could not determine long-term changes of MetS. Longer sustained changes could be associated with even higher or lower risk of laryngeal cancer. Second, this study did not have detailed biochemical information about cancer stage, laryngoscopic finding, family history, or medication history. Third, follow-up was relatively short considering the time needed for laryngeal cancer occurrence. Finally, Residual confounding by dietary factors or other risk factors as exposure to radiation or chemicals is also plausible but speculative based on our current knowledge of established risk factors for MetS and laryngeal cancer. However, the higher risk of laryngeal cancer could be explained by a change in MetS status that affected the progression or worsening of laryngeal cancer.

In conclusion, this study reaffirmed that MetS could increase the risk of laryngeal cancer. Due to the impact of MetS, patients with a history of MetS may require more rigorous screening tests for laryngeal cancer than the general population. Strategies to improve MetS may help prevent laryngeal cancer occurrence.
